# Association of apelin and apelin receptor with the risk of coronary artery disease: a meta-analysis of observational studies

**DOI:** 10.18632/oncotarget.17360

**Published:** 2017-04-21

**Authors:** Tianbao Chen, Bing Wu, Rong Lin

**Affiliations:** ^1^ Department of Cardiology, Fujian Medical University Affiliated the First Quanzhou Hospital, Quanzhou City, Fujian, People's Republic of China

**Keywords:** apelin-APLNR pathway, coronary artery disease, circulating apelin concentration, genetic polymorphism, meta-analysis

## Abstract

It is well established that apelin-APLNR (apelin receptor) pathway plays a central role in cardiovascular system. In this meta-analysis, we summarized published results on circulating apelin concentration in association with coronary artery disease (CAD), *apelin* and *APLNR* genetic polymorphism(s) in predisposition to CAD risk and circulating apelin changes after surgical treatment for CAD. The results from 15 articles were pooled. Two authors independently took charge of literature search, article selection and information collection. Overall, circulating apelin concentration was significantly lower in CAD patients (N=1021) than in controls (N=654) (weighted mean difference [WMD]: -1.285 ng/mL, 95% confidence interval [CI]: -1.790 to -0.780, *P*<0001), with significant heterogeneity (*I*^2^=99.3%) but without publication bias. For the association of *APLNR* gene rs9943582 polymorphism with CAD (patients/controls: 5975/4717), the mutant T allele was associated with a 5.2% increased risk relative to the wild C allele (odds ratio: 1.052, 95% CI: 0.990 to 1.117, *P*=0.100), without heterogeneity (*I*^2^=0.0%) or publication bias. Circulating apelin was increased significantly after surgical treatment for CAD (N=202) (WMD: 2.011 ng/mL, 95% CI: 0.541 to 3.481, *P*=0.007), with significant heterogeneity (*I*^2^=98.0%). Stratified analyses showed that circulating apelin was significantly reduced in studies with age- and sex-matched patients and controls (WMD: -1.881 ng/mL, 95% CI: -2.457 to -1.304, *P*<0.001) and with total sample size ≥125 (WMD: -1.657 ng/mL, 95% CI: -2.378 to -0.936, *P*<0.001), relative to studies without matching reports and with total sample size <125. In brief, our results suggested that circulating apelin was a prominent athero-protective marker against the development of CAD.

## INTRODUCTION

As released by the World Health Organization, coronary artery disease (CAD) is entitled as the world's biggest killer, contributing to approximately 8.76 million deaths in 2015, up from 5.74 million deaths in 1990 [1], and it will continue to be the single largest cause of disease burden in many countries worldwide by the year 2020 [2]. Several risk factors have been proposed to play a part in the development of CAD, such as age, sex, smoking, hypertension, diabetes mellitus and high-density lipoprotein cholesterol [3, 4]. However, conventional risk profiles cannot fully account for the process of coronary artery atherosclerosis, the underlying mechanism of CAD [5]. Recent years have come with major achievements in identifying a large panel of novel biomarkers [6, 7], such as tenascin-C [8] and LRP8 [9], whereas current prospects are far from encouraging, with no consensus on their medical implications. It is undoubtedly necessary to unfold a more comprehensive picture for certain promising markers affecting CAD based on the literature.

In the present study, we were intended to focus on a promising pathway containing apelin and apelin receptor (APLNR). Apelin, a peptide isolated from bovine stomach extracts, is the endogenous ligand for orphan receptor APLNR [10]. The biological plausibility of apelin-APLNR pathway in cardiovascular system is obvious, echoing some observations from clinical, epidemiologic, genetic, *in-vivo* and *in-vitro* studies. For instance, Japp et al in a clinical trial found that acute apelin administration in humans can evoke peripheral and coronary vasodilatation and raise cardiac output [11]. In a mouse model, apelin administration was found to be able to ameliorate high fat diet-induced cardiac hypertrophy and contractile dysfunction [12]. Several epidemiologic studies in humans have proposed circulating apelin as a promising predictor for CAD [13–17]; however, a systematic investigation is currently lacking. It is reasonable to hypothesize that apelin-APLNR pathway plays a central role in cardiovascular system. To fill this gap of knowledge, we summarized the results from published articles on the difference in circulating apelin concentration between CAD patients and controls through a comprehensive meta-analysis. Meanwhile, we summarized the results on *apelin* and *APLNR* genetic polymorphisms in predisposition to CAD risk. What's more, circulating apelin changes after surgical treatment for CAD were also summarized to seek additional supporting evidence.

## RESULTS

The flow diagram of search strategy and study selection for this meta-analysis is presented in Supplementary Figure S1.

### Study characteristics

Table 1A, 1B and 1C jointly show the baseline characteristics of 9 articles that assessed circulating apelin concentration between CAD patients and controls [13, 15, 16, 18–23]. As 2 articles provided the results by CAD subtypes [16, 18] and 1 article by sex [23], there were 13 studies in pooled analysis, including 1021 CAD patients and 654 controls. Year of publication ranged from 2010 to 2016. Six studies were conducted in Asian countries, 4 studies in European countries and 3 studies in Middle Eastern countries. Six studies reported circulating apelin changes in CAD patients, 4 studies in acute coronary syndrome (ACS) patients and 3 studies in acute myocardial infarction (AMI) patients. Apelin concentration was measured in plasma by 7 studies and in serum by 6 studies. Age and sex were reported to be matched between CAD patients and controls by 6 studies. Total sample size ranged from 7 to 196 in patients, and from 27 to 171 in controls.

**Table 1A:** The baseline characteristics of eligible studies for circulating apelin changes between CAD patients and controls

First author	Year	Country	Endpoint	Diagnosis	Match	Sample size	Apelin		
							Source	ELISA kit Co.	C. (ng/mL)
Kadoglou (UA)	2010	Greece	ACS	Electrocardiogram	age, sex	80/72	Serum	NA	0.60/2.99
Kadoglou (AMI)	2010	Greece	AMI	Electrocardiogram	age, sex	115/72	Serum	NA	0.67/2.99
Kadoglou (CAD)	2010	Greece	CAD	Angiography	age, sex	88/72	Serum	NA	1.35/2.99
Ye (AMI)	2012	China	AMI	Angiography	NA	87/39	Plasma	Phoenix Pharmaceuticals	2.21/2.60
Ye (CAD)	2012	China	CAD	Angiography	NA	33/39	Plasma	Phoenix Pharmaceuticals	2.54/2.60
Zhou	2014	China	ACS	Arteriography	age, sex	196/171	Plasma	Phoenix Pharmaceuticals	0.54/3.22
Akcilar	2015	Turkey	CAD	Angiography	age, sex	158/118	Plasma	Cusabio Biotech	6.13/7.64
Bilik	2015	Turkey	CAD	Angiography	NA	26/28	Serum	Phoenix Pharmaceuticals	0.18/0.65
Grzywocz	2015	Poland	AMI	Echocardiography	age, sex	104/27	Serum	NA	0.99/1.64
Pang	2015	China	CAD	Arteriography	NA	35/40	Plasma	Phoenix Pharmaceuticals	1.37/4.40
Gupta (M)	2016	India	ACS	Electrocardiogram	NA	53/60	Plasma	USCN Life Science	1.18/1.52
Gupta (F)	2016	India	ACS	Electrocardiogram	NA	7/58	Plasma	USCN Life Science	0.79/2.16
Riazian	2016	Iran	CAD	Angiography	NA	39/41	Serum	Cusabio Biotech	0.37/0.29

**Table 1B:** The baseline characteristics of eligible studies for circulating apelin changes between CAD patients and controls

First author	Age (yrs)	Males	BMI (kg/m^2^)	Hypertension	Diabetes mellitus	Family history of CAD	Smoking
Kadoglou (UA)	65.5/60.1	0.675/0.653	28.3/27.7	0.475/0.000	0.250/0.000	0.000/0.000	0.675/0.222
Kadoglou (AMI)	63.2/60.1	0.774/0.653	28.9/27.7	0.487/0.000	0.339/0.000	0.000/0.000	0.617/0.222
Kadoglou (CAD)	68.4/60.1	0.773/0.653	30.4/27.7	0.523/0.000	0.261/0.000	0.000/0.000	0.295/0.222
Ye (AMI)	69.0/66.0	0.770/0.769	24.1/24.5	0.805/0.846	0.000/0.000	NA/0.000	0.000/0.000
Ye (CAD)	64.0/66.0	0.727/0.769	24.3/24.5	0.879/0.846	0.000/0.000	NA/0.000	0.000/0.000
Zhou	55.7/56.9	0.631/0.628	NA	0.368/0.000	NA	0.072/0.000	0.523/0.514
Akcilar	64.2/61.5	0.741/0.542	NA	NA	NA	NA	NA
Bilik	53.6/51.6	0.731/0.643	28.1/26.7	0.577/0.428	0.154/0.214	NA	0.500/0.428
Grzywocz	62.0/63.0	0.740/0.296	27.1/28.5	0.78/0.93	NA	0.000/0.000	0.440/0.330
Pang	68.1/67.4	0.486/0.475	NA	1.000/0.000	0.000/0.000	NA	0.000/0.000
Gupta (M)	46.9/47.1	1.000/1.000	24.1/22.1	NA/0.000	NA/0.000	0.000/0.000	0.585/0.450
Gupta (F)	54.1/39.9	0.000/0.000	26.0/24.7	NA/0.000	NA/0.000	0.000/0.000	0.143/0.017
Riazian	66.6/70.0	0.564/0.415	30.5/29.7	0.846/0.902	0.359/0.366	0.000/0.000	NA
Riazian	66.6/70.0	0.564/0.415	30.5/29.7	0.846/0.902	0.359/0.366	0.000/0.000	NA

**Table 1C:** The baseline characteristics of eligible studies for circulating apelin changes between CAD patients and controls

First author	FBG (mg/dL)	Fasting insulin (mU/L)	TG (mg/dL)	TC (mg/dL)	HDLC (mg/dL)	LDLC (mg/dL)	HOMA-IR	Cr (μmol/L)
Kadoglou (UA)	153.5/97.3	11.85/5.78	149.2/140.2	189.8/206.5	42.5/47.4	122.6/131.1	4.49/1.39	NA
Kadoglou (AMI)	164.3/97.3	12.42/5.78	141.3/140.2	189.8/206.5	42.8/47.4	119.6/131.1	5.04/1.39	NA
Kadoglou (CAD)	134.7/97.3	7.82/5.78	147.5/140.2	179.9/206.5	45.4/47.4	109.3/131.1	1.49/1.39	NA
Ye (AMI)	NA	NA	160.2/134.5	NA	NA	NA	NA	NA
Ye (CAD)	NA	NA	173.5/134.5	NA	NA	NA	NA	NA
Zhou	NA	NA	NA	NA	NA	NA	NA	99.5/98.7
Akcilar	NA	NA	NA	NA	NA	NA	NA	NA
Bilik	106.4/124.6	NA	145.7/184.8	196.1/195.4	41.2/40.9	128.7/113.4	NA	70.7/70.7
Grzywocz	111.0/93.0	NA	148.0/119.0	193.0/173.0	44.0/50.0	124.0/99.0	NA	84.9/84.0
Pang	NA	NA	NA	NA	NA	NA	NA	NA
Gupta (M)	NA	NA	NA	NA	NA	NA	NA	NA
Gupta (F)	NA	NA	NA	NA	NA	NA	NA	NA
Riazian	119.7/116.6	18.84/8.94	159.2/147.1	171.5/158.4	37.1/43.1	104.4/85.7	5.60/2.90	NA
Riazian	119.7/116.6	18.84/8.94	159.2/147.1	171.5/158.4	37.1/43.1	104.4/85.7	5.60/2.90	NA

Abbreviations: UA, unstable angina; AMI, acute myocardial infarction; CAD, coronary artery disease; ACS, acute coronary syndrome; M, males; F, females; NA, not available; C., apelin concentration; BMI, body mass index; FBG, fasting blood glucose; TG, triglyceride; TC, total cholesterol; HDLC, high-density lipoprotein cholesterol; LDLC, low-density lipoprotein cholesterol; HOMA-IR, homeostatic model assessment for insulin resistance; Cr, creatinine. Apelin, age, males, BMI, hypertension, diabetes mellitus, family history of CAD, smoking, FBG, insulin, TG, TC, HDLC, LDLC, HOMA-IR and Cr were expressed as mean or per cent in patients/controls.

Table 2A and 2B jointly show the baseline characteristics of 3 articles that assessed the association of *APLNR* gene rs9943582 polymorphism with CAD risk [24–26]. As 1 study provided the results by sex [25], 1 study by both sex and blood pressure [26] and 1 study by both ethnicity and CAD subtype [24], there were 10 studies in pooled analysis, including 5975 CAD patients and 4717 controls. Year of publication ranged from 2009 to 2015. All 10 studies were conducted in Asian countries (6 in China, 2 in Korea and 2 in Japan). Six studies reported the association of rs9943582 with CAD risk, 2 studies with MI risk and 2 studies with risk of angina pectoris. Likewise, 6 studies had rs9943582 genotyped by PCR-related methods and 4 studies by TaqMan technique. Age and sex was reported to be matched between patients and controls by only 2 studies. Total sample size ranged from 46 to 1056 in patients, and from 349 to 1275 in controls.

**Table 2A:** The baseline characteristics of eligible studies for *APLNR* gene rs9943582 polymorphism in association with CAD risk

First author	Year	Country	Endpoint	Genotyping	Match	Sample size	Smoking	Age (yrs)	Males	SBP (mmHg)	DBP (mmHg)
Hinhara (J-MI)	2009	Japan	MI	TaqMan	NA	575/1275	0.739/NA	59.3/39.0	0.836/0.559	NA	NA
Hinhara (J-AP)	2009	Japan	AP	TaqMan	NA	46/1275	NA	65.3/39.0	0.755/0.559	NA	NA
Hinhara (K-MI)	2009	Korea	MI	TaqMan	NA	490/693	0.714/NA	61.8/57.7	0.816/0.644	NA	NA
Hinhara (K-AP)	2009	Korea	AP	TaqMan	NA	368/693	0.593/NA	63.1/57.7	0.698/0.644	NA	NA
Jin (M)	2012	China	CAD	PCR-LDR	age, sex	533/359	NA	60.7/63.0	1.000/1.000	135.2/137.0	81.2/82.5
Jin (F)	2012	China	CAD	PCR-LDR	age, sex	461/349	NA	66.0/65.1	0.000/0.000	139.5/137.4	80.4/82.6
Wang (M)	2015	China	CAD	PCR-HRM	NA	1056/525	0.338/0.256	61.6/63.7	1.000/1.000	136.0/126.0	92.0/89.0
Wang (F)	2015	China	CAD	PCR-HRM	NA	695/414			0.000/0.000		
Wang (High BP)	2015	China	CAD	PCR-HRM	NA	1030/525			0.601/0.606		
Wang (Normal BP)	2015	China	CAD	PCR-HRM	NA	721/497					

**Table 2B:** The baseline characteristics of eligible studies for *APLNR* gene rs9943582 polymorphism in association with CAD risk

First author	BMI (kg/m^2^)	TG (mg/dL)	TC (mg/dL)	HDLC (mg/dL)	LDLC (mg/dL)	FBG (mg/dL)	Cr (μmol/L)	P: CC/CT/TT	C: CC/CT/TT
Hinhara (J-MI)	23.6/NA	NA	NA	NA	NA	NA	NA	271/250/54	636/505/131
Hinhara (J-AP)	NA	NA	NA	NA	NA	NA	NA	20/22/4	636/505/131
Hinhara (K-MI)	24.6/NA	NA	NA	NA	NA	NA	NA	228/204/58	351/280/62
Hinhara (K-AP)	25.1/NA	NA	NA	NA	NA	NA	NA	180/158/30	351/280/62
Jin (M)	25.7/25.3	162.0/157.5	169.0/169.1	39.5/44.9	102.9/99.5	110.7/94.3	97.6/90.4	312/186/35	207/128/24
Jin (F)	25.0/24.9	175.2/155.8	186.8/186.9	47.6/51.9	110.3/110.3	110.5/97.4	75.5/72.5	273/152/36	195/124/30
Wang (M)	NA	169.9/160.2	170.5/164.0	42.6/46.4	104.5/93.7	NA	NA	594/382/80	336/223/49
Wang (F)	NA							356/271/68	230/159/25
Wang (High BP)	NA							577/361/92	302/183/40
Wang (Normal BP)	NA							373/292/56	264/199/34

Abbreviations: J-MI, myocardial infarction in Japanese; J-AP, angina pectoris in Japanese; K-MI, myocardial infarction in Koreans; K-AP, angina pectoris in Koreans; M, males; F, females; BP, blood pressure; CAD, coronary artery disease; SBP, systolic blood pressure; DBP, diastolic blood pressure; BMI, body mass index; TG, triglyceride; TC, total cholesterol; HDLC, high-density lipoprotein cholesterol; LDLC, low-density lipoprotein cholesterol; FBG, fasting blood glucose; Cr, creatinine; NA, not available.

Supplementary Table S1 shows the baseline characteristics of 3 articles that assessed the changes of circulating apelin after surgical treatment for CAD [17, 27, 28]. As 1 article provided the results by the operation, there were 4 studies, involving 202 patients in pooled analysis. Two studies adopted primary percutaneous coronary intervention, 1 study adopted off-pump coronary artery bypass surgery and 1 study adopted on-pump coronary artery bypass surgery. Circulating apelin concentration was measured before the surgery and at the 5^th^ day after the surgery for CAD.

### Overall estimates

**Figure 1:**
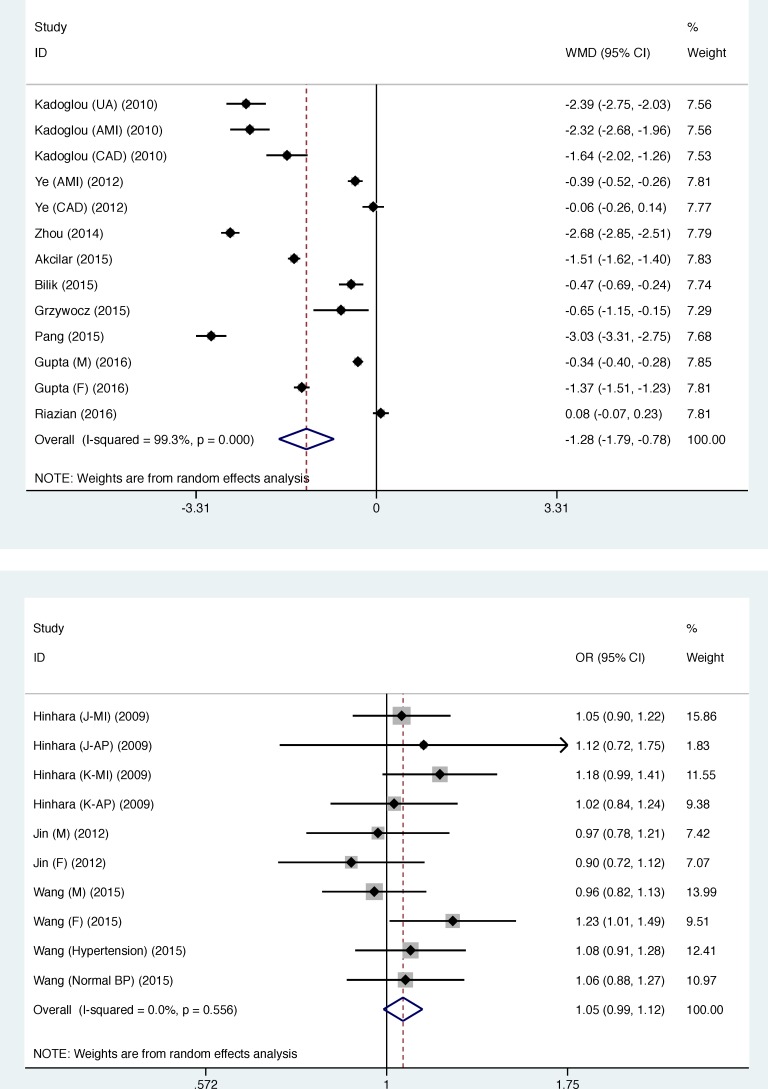
Overall weighted mean difference in circulating apelin concentration between patients with coronary artery disease (CAD) and controls (**Upper panel**), and overall prediction of APLNR gene rs9943582 polymorphism for CAD risk under the allelic model (**Lower panel**). Abbreviations: WMD, weighted mean difference; OR, odds ratio; 95% CI, 95% confidence interval.

**Figure 2:**
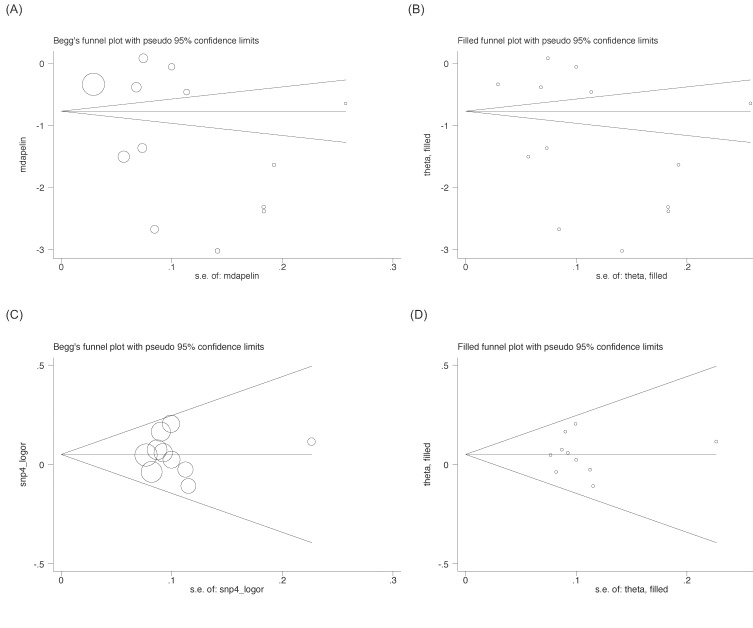
Begg’s and filled funnel plots for circulating apelin difference between patients with coronary artery disease (CAD) and controls (**A** and **B**) and the prediction of APLNR gene rs9943582 polymorphism for CAD risk under the allelic model (**C** and **D**).

In overall analysis, circulating apelin concentration was significantly lower in CAD patients than in controls (WMD: -1.285 ng/mL, 95% CI: -1.790 to -0.780, *P* < 0001), while statistical heterogeneity was significant (*I*^2^: 99.3%, *P* < 0.001) (Figure 1: the UPPER panel). As two studies involved controls with atrial fibrillation [13] and stable angina [21], respectively, a sensitivity analysis by excluding the two studies found the reduction in circulating apelin concentration was still significant (WMD: -1.465 ng/mL, 95% CI: -2.024 to -0.906, *P* < 0001), with significant heterogeneity (*I*^2^: 99.3%, *P* < 0.001). The cumulative and influential analyses were displayed in Supplementary Figure S2. The likelihood of publication bias was low as illustrated by both Begg's funnel plot and filled funnel plot (Figure 2A and 2B), as well as by both Begg's test (*P* = 0.428) and Egger's test (*P* = 0.187). In addition, differences of other clinical markers between CAD patients and controls were also tested, as shown in Table 3. Circulating FBG (WMD: 27.471 mg/dL, 95% CI: 6.350 to 48.591), fasting insulin (WMD: 5.748 mU/L, 95% CI: 2.682 to 8.814), TG (WMD: 11.783 mg/dL, 95% CI: 0.113 to 23.453) and HOMA-IR (WMD: 2.360, 95% CI: 0.222 to 1.498) levels were significantly higher in CAD patients than in controls, while circulating HDLC (WMD: -4.020 mg/dL, 95% CI: -5.801 to -2.239) was significantly lower. Statistical heterogeneity was nonsignificant for the comparisons of circulating TG, HDLC and Cr (*I*^2^: 18.5%, 3.2% and 0.0%, respectively). Also, there was no indication of publication bias for all other clinical markers in viewing both Begg's test and Egger's test (Table 3).

**Table 3:** Overall effect-size estimates of other clinical markers and the tests for between-study heterogeneity and publication bias

Clinical markers	Studies (N)	WMD	95% CI	*P*	*I*^2^	*P*	*P*_Begg's test_	*P*_Egger's test_
FBG	6	27.471	6.350 to 48.591	0.011	92.7%	<0.001	1.000	0.640
Fasting insulin	4	5.748	2.682 to 8.814	<0.001	98.0%	<0.001	1.000	0.320
TG	8	11.783	0.113 to 23.453	0.048	18.5%	0.283	0.536	0.973
TC	6	−5.121	−20.250 to 10.009	0.507	81.2%	<0.001	0.260	0.192
HDLC	6	−4.020	−5.801 to -2.239	<0.001	3.2%	0.396	0.707	0.686
LDLC	6	2.238	−13.434 to 17.911	0.780	86.1%	<0.001	0.133	0.149
HOMA-IR	4	2.360	0.222 to 1.498	0.031	97.9%	<0.001	1.000	0.230
Cr	3	0.670	−2.574 to 3.914	0.686	0.0%	0.981	NA	NA

Abbreviations: WMD, weighted mean difference; 95% CI, 95% confidence interval; FBG, fasting blood glucose; TG, triglyceride; TC, total cholesterol; HDLC, high-density lipoprotein cholesterol; LDLC, low-density lipoprotein cholesterol; HOMA-IR, homeostatic model assessment for insulin resistance; Cr, creatinine; NA, not available.

Based on all eligible articles, four polymorphisms, two in *apelin* gene (rs3761581 and rs56204867) and two in *APLNR* gene (rs948847 and rs9943582) were summarized in association with CAD risk. Considering the small number of studies for polymorphisms rs3761581 (*N* = 3), rs56204867 (*N* = 3) and rs948847 (*N* = 2), with the null association with CAD risk, only genotype distributions of rs9943582 (*N* = 10) were presented and compared between CAD patients and controls (Table 2B). No studies reported significant deviations from the Hardy-Weinberg equilibrium for rs9943582 polymorphism in control groups at a significance level of 5%.

For the association of *APLNR* gene rs9943582 polymorphism with CAD risk, the mutant T allele was associated with a 5.2% increased risk relative to the wild C allele (OR: 1.052, 95% CI: 0.990 to 1.117, *P* = 0.100) (Figure 1: the LOWER panel), and there was no observable heterogeneity (*I*^2^: 0.0%, *P* = 0.556) and a low probability of publication bias (Figure 2C and 2D) (*P* for Begg's test: 0.721 and *P* for Egger's test: 0.953). Circulating apelin concentration was increased after coronary operation (WMD: 2.011 ng/mL, 95% CI: 0.541 to 3.481, *P* = 0.007), but with strong evidence of heterogeneity (*I*^2^: 98.0%, *P* < 0.001) (data not shown).

### Stratified estimates

In attempting to seek possible explanations of significant heterogeneity, stratifying studies that assessed circulating apelin concentration between CAD patients and controls by continent, endpoint, apelin source, matching status, sample size, obesity and hypertension were conducted, respectively (Table 4). By continent, circulating apelin concentration was significantly reduced in patients relative to controls from European (WMD: -1.767 ng/mL, *P* < 0.001) and Asian (WMD: -1.307 ng/mL, *P* = 0.002) countries, while no significance was detected in populations from Middle Eastern countries. By clinical endpoint, significant reduction in circulating apelin concentration was found in ACS (WMD: -1.690 ng/mL, *P* = 0.005) or CAD (WMD: -1.099 ng/mL, *P* = 0.011) patients when compared with controls, and only marginally significant reduction was found in AMI patients. By apelin source, circulating apelin concentration was reduced significantly when measured in serum (WMD: -1.226 ng/mL, *P* = 0.007) and in plasma (WMD: -1.336 ng/mL, *P* < 0.001). By matching status for age and sex, the reduction magnitude in circulating apelin concentration was stronger in studies with matched patients and controls (WMD: -1.881 ng/mL, *P* < 0.001) than those without matching reports. Finally, median cut-off value based on total sample size of all eligible studies was 125, and circulating apelin concentration was reduced with greater magnitude for studies with total sample size ≥ 125 (WMD: -1.657 ng/mL, *P* < 0.001) than those with total sample size < 125 (WMD: -0.857, *P* = 0.007). By obesity status in controls, the reduction in circulating apelin concentration was comparable between studies with normal weight controls (BMI < 25 kg/m^2^) (WMD: -1.336 ng/mL, *P* < 0.001) and obese controls (BMI ≥ 25 kg/m^2^) (WMD: -1.226 ng/mL, *P* = 0.007). By hypertension status in controls, the reduction was even stronger in the studies involving normotensive controls (WMD: -1.964 ng/mL, *P* < 0.001) than the other studies (WMD: -1.266 ng/mL, *P* = 0.042).

**Table 4:** Weighted mean difference in circulating apelin concentration between CAD patients and controls in stratified analyses

Characteristics	Subgroups	Studies (N)	Sample size*	WMD	95% CI	*P*
Continent	Europe	4	387/243	−1.767	−2.466 to -1.069	<0.001
	Asia	6	411/407	−1.307	−2.128 to -0.487	0.002
	Middle East	3	223/187	−0.633	−1.716 to 0.450	0.252
Clinical endpoint	ACS	4	336/361	−1.690	−2.880 to -0.501	0.005
	AMI	3	306/138	−1.118	−2.390 to 0.150	0.085
	CAD	6	379/338	−1.099	−1.950 to -0.248	0.011
Apelin source	Serum	6	452/312	−1.226	−2.112 to -0.340	0.007
	Plasma	7	569/525	−1.336	−2.040 to -0.632	<0.001
Matched status	age, sex	6	741/532	−1.881	−2.457 to -1.304	<0.001
	NA	7	280/305	−0.788	−1.296 to -0.279	0.002
Sample size	≥ 125	7	828/571	−1.657	−2.378 to -0.936	<0.001
	< 125	6	193/266	−0.857	−1.482 to -0.232	0.007
Obesity**	BMI < 25 kg/m^2^	4	180/196	−1.336	−2.040 to 0.632	<0.001
	BMI ≥ 25 kg/m^2^	6	452/312	−1.226	−2.112 to -0.340	0.007
Hypertension**	Normotensive controls	7	574/545	−1.964	−2.904 to -1.023	<0.001
	Others	5	289/174	−1.266	−1.813 to -0.719	0.042

Abbreviations: ACS, acute coronary syndrome; AMI, acute myocardial infarction; CAD, coronary artery disease; BMI, body mass index; WMD, weighted mean difference; 95% CI, 95% confidence interval; NA, not available. *Sample size was expressed in CAD patients/controls. **Obesity and hypertension were determined in control groups.

## DISCUSSION

This meta-analysis summarized the results from 15 published articles with the purpose of assessing circulating apelin concentration between CAD patients and controls, the prediction of *APLNR* gene rs9943582 polymorphism for CAD risk and circulating apelin changes after surgical treatment for CAD. Our meta-analytical results collectively suggested that circulating apelin was a prominent athero-protective marker against the development of CAD. As far as we know, this is so far the first meta-analysis that systematically explores the association of apelin and APLNR, either circulating concentration change or genetic variation, with CAD risk by reviewing the current literature.

Previous studies have suggested that circulating apelin was correlated directly with obesity, and in a broader sense, with obesity-related cardiovascular events [29–31]. Apelin when binding to APLNR may regulate cardiovascular system through several mechanisms. A key mechanism with multiple consequences involves the stimulation of heart rate, the contractility and propagation of action potential, the formation of atherosclerotic plaques, the nitric oxide-mediated vasodilatation and the vascular tone regulation *via* inhibiting the electrical activity of vasopressin-releasing neurons [13, 32–34]. The results of the present meta-analysis based on 13 independent studies confirmed the findings of the most previous studies by convincingly demonstrating that circulating apelin concentration was significantly lower in CAD patients than in controls. In spite of unaccountable heterogeneity between studies, our results were less likely to be confounded by the presence of publication bias, as illustrated by both funnel plots and associated tests. In particular, the magnitude of circulation apelin reduction was remarkably potentiated in the studies with age- and sex-matched patients and controls, as well as in the studies with total sample size ≥ 125, which added to the credibility of our overall observations. Moreover, in view of divergent tendencies for circulating apelin concentration across continental populations, it can be speculated that the regulatory role of apelin in cardiovascular system, if potential involvement, might be contingent on lifestyle or environmental exposure [35, 36]. Unfortunately, the possible interactions between circulating apelin and lifestyles or environmental factors were not systematically assessed in all eligible studies, and thus it is beyond the scope of our work. It is hence expected that future efforts should focus in greater details on these promising interactions.

Besides circulating apelin difference, we also examined the susceptibility of *apelin* and *APLNR* genetic polymorphism(s) to CAD risk. Considering the statistical power, only a promoter polymorphism in *APLNR* gene, rs9943582, was summarized and presented. The mutant allele of this polymorphism was found to confer a marginally increased risk for CAD in overall analysis that was not subject to the disturbance of between-study heterogeneity and publication bias. It is universally believed that CAD is a multifactorial polygenic disease [37, 38], and the increased risk conferred by a single allele in *APLNR* gene is extremely small [25]. Although current knowledge upon the genetic underpinnings of CAD is still far from being complete, it is essential to narrow down genetic variants that could be translated to routine clinical practice. We agree that the fine-mapping of *apelin* and *APLNR* genetic defects is encouraged to aid in unveiling the pathogenesis of CAD.

Evidence from circulating apelin changes between CAD patients and controls cannot determine causality, as the changes could be secondary to CAD process itself rather than declaring a causal role. To shed some light on this issue, we additionally summarized the changes of circulating apelin concentration after surgical treatment for CAD, and our results on the basis of 4 studies revealed that circulating apelin concentration was increased remarkably at the 5^th^ day of post-surgery relative to that before the surgery, which can, at least in part, support a causal role of circulating apelin in the aetiology of CAD. Nevertheless, because our results are based on only a small number of studies, it is undoubted that larger studies should be performed to confirm the causal relevance between circulating apelin and CAD in the future.

### Study limitations

Several limitations of the present study should be acknowledged. As always, selection bias inevitably arises from a meta-analysis, especially when search scope is restricted to the English-language journals only [39]. However, as illustrated by both Begg's and filled funnel plots, as well as both Begg's and Egger's tests, the likelihood of publication bias was low for overall comparisons. In addition, as with the majority of meta-analyses, we in this study did not have access to individual level data, and we were not able to adjust for some established confounding factors (such as obesity) and assess the possible gene-environment interactions. Moreover, although this is the first meta-analysis on the association of apelin and APLNR with CAD, some subgroup analyses carried insufficient statistical power, which limited the extrapolation of our findings. Furthermore, only circulating apelin concentration was analysed, and it will be of added interest to see its changes in coronary artery tissue. It is generally believed that meta-analysis should aim at more than simply derive an overall estimate, as dissection of between-study heterogeneity can yield some valuable information. However, in the present study, performing a wide range of stratified analyses did very little to improve the impact of between-study heterogeneity on overall comparisons. There is generally a need in genetics and epidemiology for much larger and more rigorous studies to help control heterogeneity and lower publication bias.

In conclusion, our meta-analytical results suggested that circulating apelin was a prominent athero-protective marker against the development of CAD. Pending consistent replication and validation, it is anticipated that circulating apelin can be identified as a useful biomarker to allow early detection of individuals prone to the development of CAD, and further it can be proposed as an optional treatment target among high-risk CAD patients. Moreover, further studies are warranted to explore underlying mechanisms.

## MATERIALS AND METHODS

This meta-analysis was based on observational data and it was conducted following the guidelines in the Meta-analysis Of Observational Studies in Epidemiology (MOOSE) statement [40].

### Search strategies

Online computational search was confined to four literature platforms - MEDLINE, EMBASE, Web of Knowledge and Google Scholar. Search process was independently accomplished by two authors, Tianbao Chen and Bing Wu, using the same MeSH terms acknowledged by all contributing authors. Search results were merged together, with the removal of duplicates by the EndNote X8 on the Macintosh laptop. In addition, the bibliographies of retrieved articles were also searched to make literature coverage as comprehensive as possible.

### Article selection

The full-process of selecting all potential articles was independently accomplished by two authors, Tianbao Chen and Bing Wu, based on two steps. The first step was to remove articles that were not published in the English language, examined apelin in animals or focused on the signalling cascades of apelin-APLNR pathway after reviewing the title and/or abstract. The second step was to access the full text of remaining articles to see whether the results on circulating apelin difference between CAD patients and controls, or the association of *apelin* and *APLNR* genetic polymorphism(s) with CAD risk, or circulating apelin changes after surgical treatment for CAD. In the meanwhile, population source of each qualified article was inspected to remove duplicate publications from the same research team. In case of any disagreement, a discussion between the two authors was held until reaching a consensus.

### Inclusion and exclusion criteria

Both inclusion and exclusion criteria applied to each potential article. For inclusion criteria, the clinical endpoint should be CAD and the results were available on circulating apelin concentration or genotype distributions of polymorphism(s) in *apelin* and *APLNR* genes between CAD patients and non-CAD controls, as well as circulating apelin changes after surgical treatment for CAD. Exclusion criteria embraced the publication types in either abstract or poster presentation of international meetings, case report, editorial, letter to the editor, narrative or quantitative reviewer, as well as the investigations on CAD severity or response to drug treatment.

### Information collection

The same two authors, Tianbao Chen and Bing Wu, independently collected the results of interest from each qualified article, including surname of the first author, year of publication, area where study subjects resided in, CAD subtype, diagnostic criteria of both CAD patients and controls, source of controls, apelin assaying method, circulating apelin source, matching status between patients and controls, sample size of each group, circulating apelin concentration between CAD patients and controls or the association of *apelin* and *APLNR* genetic polymorphisms with CAD risk or circulating apelin changes after surgical treatment for CAD, as well as the demographic and clinical characteristics such as age, sex, body mass index (BMI), the per cents of hypertension, diabetes mellitus and family CAD history, systolic blood pressure (SBP), diastolic blood pressure (DBP), smoking status and mean levels of circulating triglyceride (TG), total cholesterol (TC), high-density lipoprotein cholesterol (HDLC), low-density lipoprotein cholesterol (LDLC), fasting blood glucose (FBG), fasting insulin, homeostatic model assessment for insulin resistance (HOMA-IR) and creatinine (Cr) in both CAD patients and controls, if appropriate. Information collected by the two authors was computationally compared for accuracy, and any disagreement was checked and solved with consensus.

### Statistical analysis

Mean differences of demographic and clinical characteristics were compared between patients and controls using the unpaired t test or the Mann-Whitney U test, as appropriate. Circulating apelin changes between CAD patients and controls or after surgical treatment for CAD were expressed by using weighted mean difference (WMD) and its 95% confidence interval (95% CI), and for the prediction of polymorphism(s) in *apelin* and *APLNR* genes for CAD risk by using odds ratio (OR) and its 95% CI. The effect-size estimates were computed using the random-effects model. Statistical magnitude of between-study heterogeneity was expressed by the *I*^2^, the inconsistency index, which can be interpreted as the per cent of variability due to heterogeneity between studies rather than a sampling error. A larger *I*^2^ value indicates a higher probability of heterogeneity, and it is universally accepted that this value over 50% denotes statistically significant heterogeneity. The likely explanations of heterogeneity were assessed by stratified analyses. Cumulative analysis and influential analysis were also presented. Publication bias, a type of bias referring to the decreased probability of studies’ results being published when they are near the null, not statistically significant or otherwise less interesting [41], was graphically illustrated by both Begg's funnel plot and filled funnel plot, and was statistically justified by both Begg's test and Egger's linear regression test.

Data management and statistical analyses were accomplished with the aid of packages in the STATA/SE software (version 14.0, StataCorp, Texas, USA) on the Macintosh laptop.

## SUPPLEMENTARY MATERIALS FIGURES AND TABLES


