# Supplementary material for: Association of apelin and apelin receptor with the risk of coronary artery disease: a meta-analysis of observational studies
Source: Oncotarget. 2017 Apr 21;8(34):57345–55. doi: 10.18632/oncotarget.17360 (PMC5593646; doi:10.18632/oncotarget.17360)
Supplement: Supplementary file 1 [file oncotarget-08-57345-s001.pdf]

Association of apelin and apelin receptor with the risk of coronary artery disease: a meta-analysis of observational studies

Supplementary Material

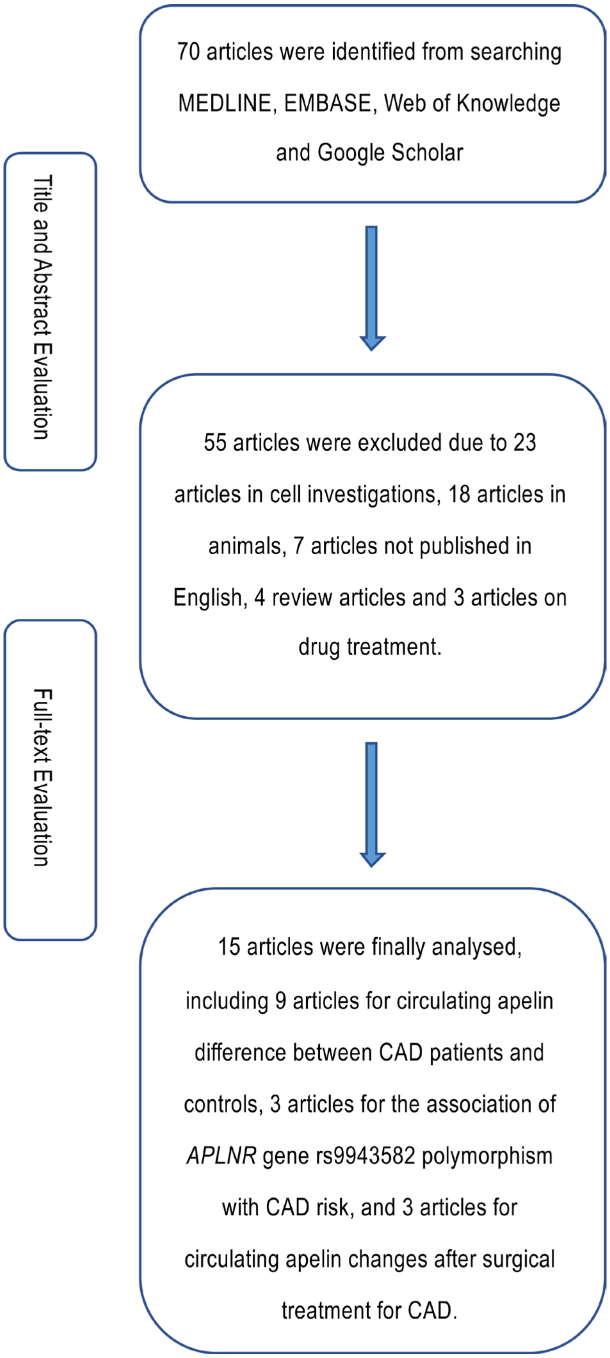

Supplementary Figure S1: Flow diagram of search strategy and study selection

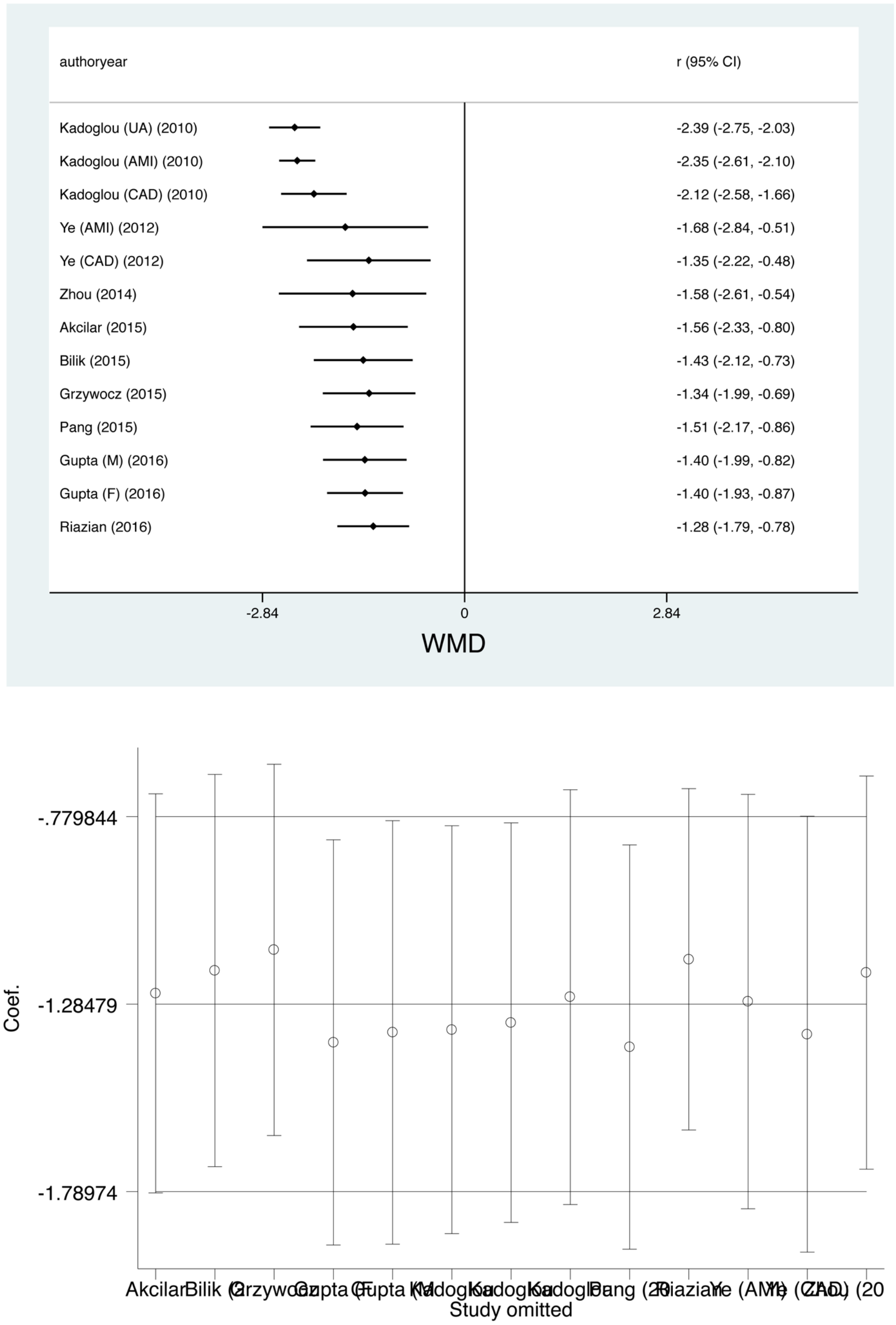

Supplementary Figure S2: Cumulative analysis and influential analysis for the difference in circulating apelin concentration between patients with coronary artery disease and controls. WMD, weighted mean difference; 95% CI, 95% confidence interval.

Supplementary Table S1. The baseline characteristics of 3 eligible articles for the changes of circulating apelin after surgical treatment for coronary artery disease

First author	Year	Intervention	Sample size	Apelin (ng/mL): mean/SD	
				Pre-surgery	Post-surgery (5-day)
Mahar (OPCAB)	2008	OPCAB	22	4.60/1.07	7.70/1.50
Mahar (CCAB)	2008	CCAB	24	5.05/1.84	10.71/2.30
Kuklinska	2010	pPCI	78	2.14/0.31	2.01/1.70
Tycinska	2010	pPCI	78	0.33/0.05	0.31/0.07

Abbreviations: OPCAB, off-pump coronary artery bypass surgery; CCAB, on-pump coronary artery bypass surgery; pPCI, primary percutaneous coronary intervention; SD, standard deviation.
